# Fatty Acid Synthesis in Glial Cells of the CNS

**DOI:** 10.3390/ijms22158159

**Published:** 2021-07-29

**Authors:** Aida V. Garcia Corrales, Mansour Haidar, Jeroen F. J. Bogie, Jerome J. A. Hendriks

**Affiliations:** Department of Immunology and Infection, Biomedical Research Institute, Hasselt University, 3590 Diepenbeek, Belgium; aida.garciacorrales@uhasselt.be (A.V.G.C.); mansour.haidar@uhasselt.be (M.H.); jeroen.bogie@uhasselt.be (J.F.J.B.)

**Keywords:** fatty acids, glia, central nervous system, neurological disease, CNS repair

## Abstract

Fatty acids (FAs) are of crucial importance for brain homeostasis and neural function. Glia cells support the high demand of FAs that the central nervous system (CNS) needs for its proper functioning. Additionally, FAs can modulate inflammation and direct CNS repair, thereby contributing to brain pathologies such Alzheimer’s disease or multiple sclerosis. Intervention strategies targeting FA synthesis in glia represents a potential therapeutic opportunity for several CNS diseases.

## 1. Introduction

The central nervous system (CNS) has an exceptionally high lipid content. In fact, lipids constitute about half of the brain tissue dry weight, making it the second most lipid rich organ after adipose tissue [1]. In addition, the brain contains the highest diversity of lipids than any other organ. Fatty acids (FA) are essential monomeric components that define the structural diversity of lipids and determine their functional properties in the CNS [2]. FAs and their metabolites are critical for brain homeostasis and influence many neural functions, including cell survival, neurogenesis and synaptogenesis [3,4]. Glial cells are a highly heterogeneous population of cells and predominate the mammalian brain [5]. Astrocytes, oligodendrocytes and microglia are the major types of glial cells in the CNS [6]. Their main function is to sustain a homeostatic environment for neuronal circuits, providing not only structural or trophic support but also controlling neuronal function and plasticity [7,8,9]. To do so, glial cells heavily rely on transient and temporal changes in the FA and lipid metabolism [10]. The critical role of lipids in CNS physiology and cell signaling has been validated by the many neurological disorders neurodegenerative diseases such as Alzheimer’s, Parkinson’s or Niemann-Pick, where lipid metabolism is dysregulated [11,12,13]. Previously, the synthesis of other lipid species such cholesterol by glial cells and their effect in neurological diseases have been extensively described [14,15,16,17,18]. In this review, we elaborate on the current knowledge of FA synthesis and processing pathways in glial cells as well as the crosstalk of FA synthesis and glia in CNS pathology. We further discuss how modulating these processes may offer novel therapeutic options for various neurological disorders.

## 2. Fatty Acid Biosynthesis in Healthy CNS

### 2.1. De Novo Fatty Acid Synthesis

**Figure 1:**
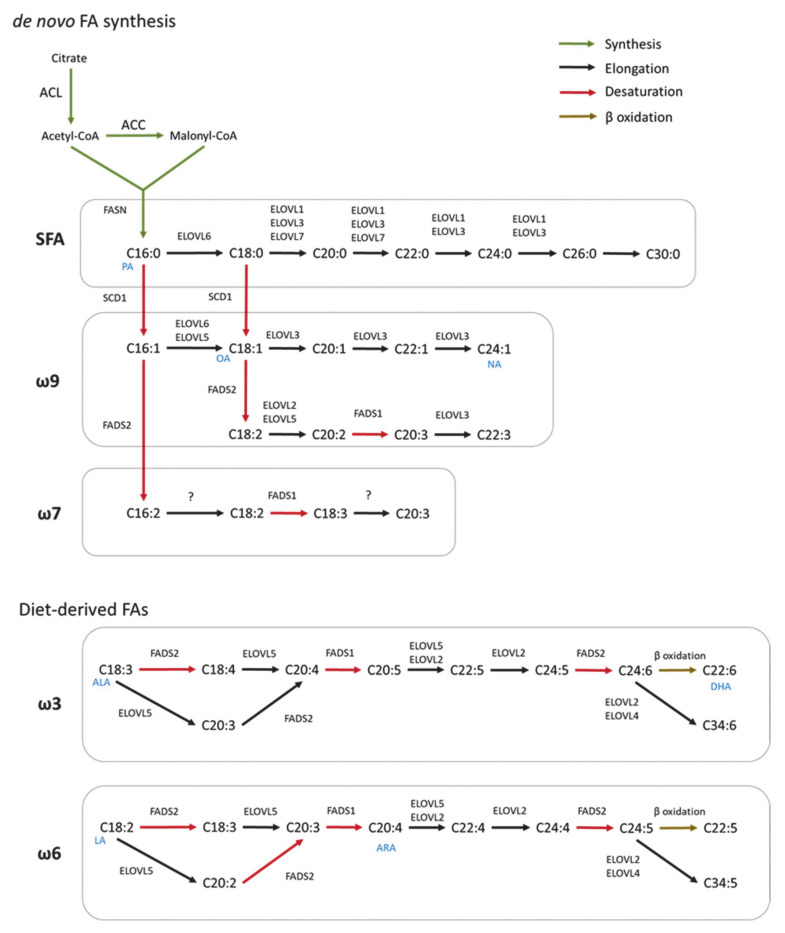
Scheme of FA synthesis in mammals. FAs are elongated (ELOVL1–7) and/or desaturated (SCD, FADS) to generate complex FAs. Long chain saturated FAs (LCSFA) and unsaturated FAs of ω9 and ω7 can be synthesized from palmitic acid (PA, C16:0) produced by the *de novo* FA synthesis. Long-chain unsaturated FAs of the ω6 and ω3 series can only be synthesized from essential diet-derived FAs (OA, oleic acid; NA, nervonic acid; ALA, α-linolenic acid; DHA, docosahexaenoic acids; LA, linoleic acid; ARA, arachidonic acid).

*De novo* FA synthesis is a highly conserved process between bacteria and eukaryotes [19]. In mammals, this process is a critical anabolic pathway that takes place in the cytoplasm [20]. The mechanism of FA synthesis consists of a coordinated series of enzymatic reactions that sequentially extend an alkanoic chain by a series of decarboxylative condensation reactions (Figure 1) [21]. Initially, citrate is converted to acetyl-CoA by ATP-citrate lyase (ACLY). The resulting acetyl-CoA is carboxylated to yield malonyl-CoA by acetyl-CoA carboxylase (ACC). Then, Acetyl-CoA and malonyl-CoA are coupled to the acyl-carrier protein domain of the rate-limiting enzyme fatty acid synthase (FASN) [22]. The elongation of the chain occurs by repeating the condensation cycle until a 16-carbon palmitic acid (16:0) is generated. Next, palmitic acid is further elongated and desaturated to generate complex fatty acids. Alternatively, *de novo* synthesis also occurs in mitochondria but this pathway closely resembles the prokaryotic FA synthesis pathway [23]. However, this pathway has only one known product, lipoic acid, which functions as a cofactor for several important mitochondrial multienzyme complexes [24].

### 2.2. Elongation

In mammals, FA elongation depends on a set of enzymes termed elongation of very long-chain fatty acid enzymes (ELOVLs) [25]. ELOVLs are located in the endoplasmic reticulum (ER) and catalyze FA elongation via the condensation of a malonyl-CoA to an acyl-CoA molecule to yield 3-ketoacyl-CoA, which is the first rate limiting step in the elongation cycle of FAs [26]. Then, 3-ketoacyl-CoA is reduced and dehydrated to produce trans-2-enoyl-CoA, which is finally reduced to form the elongated acyl-CoA. To date, seven ELOVLs have been identified in mammals (ELOVL1–7) (Figure 1). ELOVL1, 5, 6 and 7 are ubiquitously expressed, while ELOVL2, 3 and 4 are tissue specific [27,28]. In terms of target substrates, ELOVL1, 3 and 6 elongate saturated (SFAs) and monounsaturated fatty acids (MUFAs), whereas ELOVL2, 4 and 5 elongate polyunsaturated fatty acids (PUFAs) [29].

### 2.3. Desaturation

FA desaturation is accomplished by the introduction of a double bond at specific positions within the FA carbon chain, a process catalyzed by Acyl-coenzyme A (CoA) desaturases [30]. Mammalian cells express Δ9, Δ6 and Δ5-desaturases in which the Δ-number specifies the position where the double bond is introduced [31]. Desaturases are divided in two families, stearoyl-coA desaturases (SCDs), and fatty acid desaturases (FADS) (Figure 1) [32]. SCDs transform SFAs into MUFAs by introducing a single double bond at position Δ9 of the FA chain. In mice, SCDs are present in four isoforms (SCD1–4) which present a distinct tissue distribution pattern [33,34,35]. However, humans only have a highly homologous gene of SCD1 that is ubiquitously expressed in all tissues [36]. FADS are enzymes that catalyze the desaturation of PUFAs. Humans express three FADS (FADS 1–3). The desaturation reactions at positions 5 and 6 of the FA chain are catalyzed by desaturases FADS1 and FADS2, respectively. The function of FADS3 has not been described but it is suggested to catalyze Δ13-desaturation [37]. Mammals lack the Δ12 and Δ15-desaturases present in plants and, consequently, they cannot synthesize FA of the ω6 and ω3 series [38]. These so-called essential fatty acids must be provided by the diet and serve as precursors for the synthesis of longer PUFAs, including arachidonic (ARA) and docosahexaenoic acids (DHA) required for many functions such as the regulation of the membrane composition and signaling pathways [32]. In addition, synthesis of DHA and docosapentaenoic acid undergo one cycle of peroxisomal β-oxidation [39]. Both, *de novo* FAs and diet-derived FA share the same enzymes for their elongation and desaturation.

## 3. Synthesis of Complex FA Species

As previously stated, FAs are the building blocks for many complex lipids. Phospholipids (PL) represent the majority of cellular FAs and are the main component of cell membranes and the major lipid class in the brain [40]. Their structure consists of a hydrophilic head group attached by a phosphodiester bridge to a hydrophobic backbone with two esterified FAs, making PLs amphipathic molecules [41]. PL are divided in two categories: glycerophospholipids and sphingolipids.

### 3.1. Glycerophospholipids

Glycerophospholipids (GPs) are the most abundant PL. They all have at least one hydrophobic chain linked to a glycerol backbone by an ester or ether linkage [42]. Phosphatidic acid serves as a precursor of most GPs, a major lipid second messenger carrying signaling information and together with its precursor lysophosphatidic acid (LPA) is involved in gliomas and brain cancers [43,44]. GPs are subdivided into distinct subclasses, based on the nature of the polar head group: phosphatidylcholine (PC), phosphatidylethanolamine (PE), phosphatidylserine (PS) and phosphatidylinositol (PI) [45]. PC, PE and PS are synthesized from phosphatidate by the cytidine diphosphate (CDP)–diacylglycerol pathway, while PC and PE can also synthesized by the Kennedy (CDP–choline and CDP–ethanolamine) pathway [46,47]. GPs synthesis takes place on the ER and the mitochondrial outer membrane [48].

GPs are essential for basic cellular functioning. PC, the most abundant phospholipid in all mammalian cell membranes, plays a vital part in membrane fusion, transport, endocytosis and enzymatic catalysis [49]. PC is an important constituent of the myelin layer and has been linked to myelination during development and repair [50]. In the brain, PC contains mostly saturated C16:0 and monounsaturated C18:1 FAs [51]. PE comprises 45% of total PL in the brain and is rich in PUFA. PE is used as a substrate for the production of endocannabinoids [52]. Endocannabinoids are lipid-based retrograde neurotransmitters that bind to cannabinoid receptors to modulate synaptic signaling [53]. PS is synthesized from phosphatidylcholine or phosphatidylethanolamine by exchanging the base head group with serine. PS contains mainly C18:0, C18:1, and DHA [54]. PS in grey matter is predominantly rich in DHA. PS is required for the activation of Akt, Raf-1 and protein kinase C signaling which are known to stimulate neuronal survival, neurite growth and synaptogenesis [55]. In addition, PS play a critical role in signal transduction pathways, including the PI3K/Akt, Raf/Ras and protein kinase C pathways, and modulation neurotransmitter release and reception. The brain contains the highest concentrations of PI among animal tissues and is mainly composed by stearic acid and ARA [54]. PI generates phosphoinositides via phosphorylation on the inositol headgroup. In the CNS, phosphoinositides modulate ion channels, affecting electrical signaling as well as calcium and neurotransmitter release [56]. In summary, GP functions are widespread and diverse in the CNS.

### 3.2. Sphingolipids

Sphingolipids are a family of bioactive molecules that comprise an essential group of lipids containing a long-chain sphingoid-base backbone [45]. The FA components of sphingolipids are usually saturated or monounsaturated, and contain 16, 18, 22, or 24 carbon atoms [57]. Sphingolipids influence structural properties of membranes and function as cell signaling modulators and mediators. [58]. This group of lipids include species such as sphingosines, ceramides and sphingosine 1-phosphate (S1P), and sphingomyelin (SM) that are involved in numerous cell signaling pathways.

*De novo* sphingolipid synthesis takes place in the ER, where the condensation of activated C_16_ FA palmitoyl-CoA and amino acid L-serine is catalyzed by serine palmitoyltransferase (SPT), forming 3-ketodihydrosphingosine. Next, 3-keto-dihydrosphingosine is reduced to dihydrosphingosine and then acetylated by one of the six (dihydro)ceramide synthases (CerS1–6) to produce ceramides. Ceramide is the core constituent of all sphingolipids. Ceramides are potent signaling molecules that control cellular physiology, ranging from apoptosis, cell differentiation and cell cycle [59]. Ceramides are then transported to the Golgi apparatus where they can be further processed into other sphingolipids such as sphingomyelin. Sphingomyelin, together with ceramides, is involved in the process of neurogenesis and synaptogenesis [60]. Besides this, sphingomyelin is an important structural component of cell membranes and a major constituent of myelin [61]. Sphingomyelin fatty acids comprise mainly stearic (C18:0), lignoceric (C24:0), and nervonic (C24:1) acids [54]. Ceramides can also be metabolized by ceramidases, that remove the amide-linked FA to form sphingosine. Then, sphingosine can be phosphorylated by sphingosine kinase 1 and 2 (SPHK1 and 2) to produce S1P [62]. S1P is recognized as a molecule regulating several processes such as neuronal growth and survival, cell motility, angiogenesis and immune cell migration [63]. In oligodendrocytes S1P is shown to promote differentiation by activating NT3 [64]. In addition, S1P receptors (S1PRs) are expressed in nearly all CNS cells including neurons and glial cells [65]. For example, in microglia S1PR2 and 3 are linked to a more inflammatory phenotype while S1PR1 drives microglia into a more anti-inflammatory phenotype [66,67]. The transmission of intracellular signals by S1PRS depends on the receptor expression in each cell [68]. All these findings indicate that sphingolipids have important structural and functional roles in cell cycle and signaling in the CNS.

## 4. Fatty Acid Synthesis in Glial Cells

### 4.1. Astrocytes

Astrocytes are the most abundant cell type in the CNS [69]. Astrocytes provide structural, functional and metabolic support for neurons, and they are also involved in neuroplasticity and interneuronal communication [70]. Astrocytes promote synaptic formation and function by producing thrombospondins and tumor necrosis factor α (TNFα), as well as synaptic elimination by releasing transforming growth factor-β (TGFβ). Additionally, astrocytes contribute to neuronal information processing by decoding GABAergic synaptic activity via the release of glutamate or ATP/adenosine [71].

**Figure 2:**
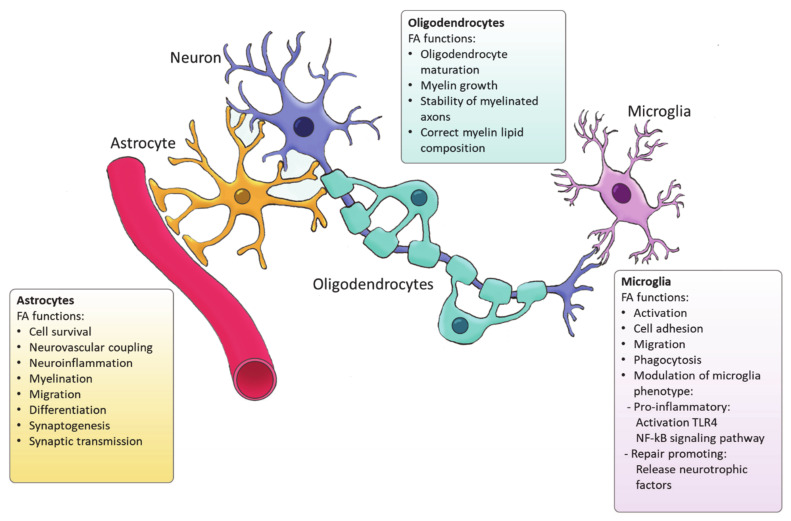
Functions of FA synthesized by glial cells.

Ample evidence indicates that FA metabolism drives the function of astrocytes in health and disease, and vice versa, astrocytes control lipid homeostasis in the CNS (Figure 2). For example, Tabernero et al. demonstrated that astrocytic FA acid synthesis is essential for neuronal differentiation during development [72]. Specifically, they reported that a developmental increase in albumin triggers the expression of sterol regulatory-element binding proteins 1 (SREBP-1) and SCD in astrocytes, resulting in the accumulation of oleic acid, a MUFA. Astrocytic oleic acid is horizontal transferred to PC and PE in neurons, enhancing neuronal differentiation and used by oligodendrocytes to synthesize sphingomyelin, stimulating myelination [72]. On that same note, astrocytic oleic acid promotes neuron migration and synaptogenesis by inducing the expression of DCX and GAP-43, respectively [73].

Astrocytes are the main source of DHA and ARA in the CNS [74] that are derived from the essential FA. In vitro and in vivo studies indicate that DHA is involved in neuronal cell growth and differentiation [75]. Although most of the DHA in the CNS is dietary derived, synthesis of DHA from α-linolenic acid by astrocytes as a response to various stimuli plays an important role in neuroinflammation and cell survival [74]. DHA attenuates microglial-induced inflammation by inhibiting the NFκB and MAPK pathway [59], while DHA deficiency increases the expression of pro-inflammatory cytokines such as IL-6, IL-1β or TNF-α [76]. DHA is crucial for the differentiation of astrocytes and inhibits ER stress [77,78]. On the other hand, ARA acts as a messenger controlling the electrical and biochemical behavior of neurons and glial cells. ARA mediates synaptic transmission via acting on most voltage-gated and ligand-gated ion channels [79]. In addition, astrocytes direct neurovascular coupling by synthesizing vasodilatory ARA derivatives [80]. Besides DHA and ARA, other FAs such palmitic acid and stearic acid are generated by astrocytes upon inflammatory stimulation [74]. Interestingly, astrocytic loss of cholesterol synthesis was found to impact whole-body metabolism via sterol regulatory element-binding protein 2 (SREBP2) modulation [81]. SREBP2 is the major transcription factor regulating cholesterol synthetic genes [82]. Astrocyte-specific SREBP2 knock out mice showed altered fat composition and metabolism. All in all, these findings demonstrate that astrocyte-derived FAs are essential for the maintenance and function or the CNS and may even impact systemic metabolism. Nevertheless, further characterization of the FA generated by astrocytes, their bioactivity as well as their relevance in regulating brain function is required.

### 4.2. Oligodendrocytes

In the CNS, oligodendrocytes are specialized glial cells responsible for producing myelin sheaths [83,84]. The myelin sheath is characterized by a high percentage of lipids (70–85%) [85]. Apart from cholesterol, all the lipids that form myelin use FAs as their fundamental structure blocks. Therefore oligodendrocytes heavily rely on FA synthesis for membrane production during myelination [86]. Among the FAs present in myelin lipids, about 80% have a chain length of 18 carbon atoms or less and 6% are PUFAs [87]. A recent study has demonstrated that *de novo* FA synthesis is critical for accurate CNS myelination. This includes radial myelin growth, stability of myelinated axons and correct myelin lipid composition (Figure 2) [88]. Even though FASN is not indispensable for the proliferation and differentiation of oligodendrocytes, FASN deficiency was proven to affect correct maturation of oligodendrocytes and myelination [88]. Moreover, myelin contains high levels of saturated very long chain fatty acids (VLCFA). VLCFAs formation is dependent on ELOVLs activity. Saturated VLCFA are essential for providing a thick permeability barrier for ions to insulate axons [89]. In the CNS, ELOVL4 expression was mainly detected in neurons, although a small group of ELOVL4 positive cells has been observed in the brain white matter, suggesting potential expression by oligodendrocytes [90]. This indicates that ELOVL4 could play a role in the production of VLCFA that form the myelin sheath. In addition, PC is an abundant phospholipid in myelin and its synthesis in oligodendrocytes depends on *de novo* synthesis through choline uptake [50]. Altogether, current evidence points to a crucial role for FA synthesis in the differentiation of oligodendrocytes and subsequent myelination However future research needs to elucidate the underlying mechanisms.

### 4.3. Microglia

Microglia are the resident immune cells of the CNS and comprise ∼10–20% of all glial cells [91]. Unlike astrocytes and oligodendrocytes, which are derived from a common lineage of neural progenitor cells, microglia are originated from yolk sac primitive macrophage progenitors that invade the brain at the very early stages of embryonic development. Nowadays, microglia are considered a versatile group of cells [92,93]. Microglia provide the first line of innate immunity of the CNS but its functions go beyond scavenging of debris and infectious agents [92]. Microglia mediate synaptic pruning [94] and regulate neurogenesis and repair [95,96]. Microglia are rich in phosphatidylglycerols and sphingomyelins, containing high levels of specific sphingomyelin which are nearly absent in other glia cells. During the last decade, several studies have demonstrated the importance of FAs in directing microglia function (Figure 2) [97]. Microglia can adopt distinctive phenotypes in response to different stimuli with the classically activated inflammatory state and the alternatively activated, inflammation resolving state as the extremes [7]. The inflammatory phenotype is characterized by the production of pro-inflammatory cytokines and neurotoxic components whereas the alternatively activated phenotype is characterized by the release anti-inflammatory and neurotrophic factors, granting them a repair promoting phenotype [98,99,100]. Yet, the phenotypes found in vivo significantly differ from these two extremes since they display a spatiotemporal spectrum of phenotypes [98,101]. Long chain SFAs, for instance palmitic and stearic acids, contribute to a pro-inflammatory phenotype by activating Toll-like receptor 4 (TLR4) and NF-kB signaling pathways [102,103,104,105]. In contrast, n-3 PUFAs stimulate an anti-inflammatory phenotype [106]. MUFAs such as oleic acid are described to promote anti-inflammatory processes via activation of the transcription factor peroxisome proliferator-activated receptor [97]. However, our recent study shows that MUFAs generated by SCD1 can shift microglia and macrophages into an inflammatory phenotype [107]. Although FAs and their derivatives are crucial for defining microglial function, only few studies describe the involvement of *de novo* FA synthesis in directing microglia activity. Despite the lack of studies in microglia, there are extensive studies on FA metabolism in macrophages. Microglia and macrophages share many features and during neuroinflammatory responses macrophages infiltrate the CNS where they, alongside microglia, execute innate effector mechanisms [108]. Therefore, it can be expected that there are parallels in the regulation of the function of both phagocyte types by FA metabolism. In macrophages, FA synthesis is indispensable for membrane remodeling and the synthesis of inflammatory factors [109]. Moreover, FASN has shown to be required for inflammatory activation of macrophages. Lack of FASN not only disrupts cell membrane composition by impairing the retention of plasma membrane cholesterol but also alters Rho GTPase trafficking, a process essential for cell adhesion, migration and activation [110]. Talamonti et al. demonstrated that ELOVL2 deficiency decreases DHA levels in macrophages, affecting their plasticity and promoting a hyperactive inflammatory phenotype [111]. In addition, microglia can synthesize neuroprotectin PD1 (NPD1) from DHA, a specialized pro-resolving lipid mediator (SPM) [112]. SPMs are a family of bioactive metabolites generated in response to inflammation by enzymatic of PUFAs [113]. NPD1 is known to promote phagocytosis and resolve inflammation [114]. Taken together, while SFA synthesis is suggested to favor inflammatory activation of microglia, PUFAs biosynthesis promotes an anti-inflammatory phenotype, and MUFA synthesis seems to have a dual influence on the microglia phenotype which probably depends on the disease context.

## 5. FA Synthesis in Neurological Diseases

FA synthesis is essential for the correct physiological function of the brain. Alteration in its metabolism has been associated to numerous neurological diseases such as Alzheimer’s disease (AD), Parkinson’s disease (PD) and Multiple Sclerosis (MS).

A growing body of evidence indicates that lipid metabolism is linked to AD pathology. AD is the most common neurodegenerative disorder and responsible for up to 70% of dementia cases. The hallmarks of AD pathology are the deposition of amyloid-β (Aβ) plaques in brain extracellular space and neurofibrillary tangles inside neurons [115]. ApoE is the main lipoprotein in the brain and participates in Aβ production, aggregation, and clearance in an isoform-dependent manner [116]. ApoE is mainly expressed in astrocytes and microglia and appears in three major isoforms, ApoE2, ApoE3, and ApoE4. ApoE4 has been identified as the most prevalent genetic risk factor for AD. In astrocytes, ApoE4 impairs FA metabolism by enhancing *de novo* FA synthesis, reducing FA degradation, and promoting the accumulation of lipid droplets [117]. AD patients have elevated levels of ARA but reduced levels of LA compared with healthy controls [118]. As stated above, astrocytes are the main source of ARA in the CNS. Activation of the ARA cascade leads to an increase in Aβ and causes impairment in working memory induced by IL-1β [119]. In microglia, ApoE4 is associated with an impairment of Aβ clearance and with the switch to a pro-inflammatory, disease-associated microglia phenotype [120]. These pro-inflammatory microglia show an upregulated FA synthesis and SFA production. Stearic acid (C18:0) concentration is significantly reduced in the frontal and temporal cortex of AD patients, whereas oleic acid (C18:1) and palmitic acid (C16:0) increase in the frontal temporal cortex and in the parietal cortex [121]. In addition, MUFAs are significantly increased in brain tissue of AD patients, which was strongly correlated with cognitive dysfunction and an increase of SCD1 transcription [122]. Overexpression of SCD1 has been linked to an increased secretion of Aβ, suggesting that inhibition of the activity of SCD1 may result in the reduction of Aβ [123]. Furthermore, astrocytes increase the synthesis of palmitic and stearic acid in AD plaques, which induce hyperphosphorylation of tau and upregulation of β-secretase [124]. The AD brain exhibits a reduction of NPD1 and DHA levels [125]. NPD1 mediates modulation of a- and b-secretase activity that results in reduced Aβ shedding [126]. In addition, DHA can downregulate secretion of A β peptides and is the precursor of NDP1. DHA and NPD1 enhance anti-inflammatory and anti-apoptotic gene expression, such Bcl-2 proteins [125]. DHA depletion in CSF fractions in AD is consistent with the importance of 3ω PUFAs in cognitive function [127]. Defects in phosphatidic acid synthesis by phospholipases D has also been reported in AD [44]. Therefore, AD is critically linked to FA metabolism and targeting *de novo* FA synthesis may provide a novel strategy to modulate AD pathology.

PD is the most frequently occurring movement disorder and the second-most common neurodegenerative disease after AD [128]. PD is characterized by neuronal loss in the substantia nigra and intracellular accumulation of α-synuclein [129]. α-synuclein is a protein present ubiquitously in all glial cells [130,131,132]. Although the molecular basis of PD is still unknown, there is increasing evidence that FA synthesis pathways are associated with PD pathology. SFA levels are present in frontal cortical lipid rafts from PD patients [133]. In the anterior cingulate cortex, there is a significant shift in the acyl chain composition of both ceramides and sphingomyelins toward shorter FA composition (C16:0, C18:0, and C18:1) [134]. PUFAs contribute to mechanisms leading to abnormal α-synuclein accumulation. Astrocytic DHA and ARA induce oligomerization and conformational changes in α-synuclein, enhancing its accumulation. Additionally, prolonged exposure to PUFA triggers the formation of α-synuclein fibrils, suggesting that FA desaturation is boosted [135]. In CSF, a significant increment was mostly observed at the level of PUFA [133,136]. Interestingly, inhibition of SCD1 enhanced the survival of human neurons in the presence of toxic α-synuclein [137]. Therefore, FA synthesis of MUFAs and PUFAs by glial cells may contribute to the exacerbation of PD pathology. These findings indicate that FAs are involved in PD pathology although further research is needed to substantiate this link.

MS is a chronic autoimmune neurodegenerative disease characterized by multiple focal demyelinated lesions accompanied by variable gliosis, inflammation and neurodegeneration [138]. Even though traditionally MS has been considered an inflammatory autoimmune disease, anti-inflammatory therapies are insufficient as the disease progresses. In MS, lipid metabolism is dysregulated, causing changes in the lipid profile in CNS and cerebrospinal fluid of patients as compared to healthy individuals [139,140,141]. Most of MS patients show a decrease of PUFA species such C18:2 or C20:4 and increase of SFA such as C20:0. SCD1 is important to generate nervonic acid (C24:1), which is the major FA component of the sphingomyelin and, therefore, essential for remyelination [142]. Conversely, our previous study has shown that SCD1 controls the inflammatory phenotype of myelin phagocytosing macrophages and microglia and thereby impacts inflammatory and repair processes [107]. SCD1 inhibition promoted a more anti-inflammatory phenotype in macrophages and microglia, resulting in an enhanced remyelination [107]. DHA was also found to regulate inflammatory responses in microglia and modulating dendritic cell-dependent T cell activation [143,144]. In addition, DHA serves as a precursor for the biosynthesis of sphingomyelin. As discussed earlier, sphingomyelin is a major myelin component and its biosynthesis is crucial for remyelination. Furthermore, FA synthesis in oligodendrocytes is essential for efficient remyelination [88,145]. In fact, an important regulator of FA synthesis, biotin, promotes myelination through enhanced myelin formation in oligodendrocytes by increasing malonyl-CoA synthesis [145]. In addition, SPMs, lipid mediators derived from essential FAs, have attracted significant interest as possible therapeutic targets due to their potent role as immunoresolvents reducing inflammation and stimulating reparative processes [146]. Significantly higher levels of the SPM NPD1 has been observed in highly active MS patients [114,147]. As previously stated, NPD1 stimulates phagocytosis and resolves inflammation, suggesting a link with the increased myelin phagocytic activity in the active lesions and the release of pro-resolving mediators. Accordingly, NPD1 levels decrease as the disease progresses. Altogether, this indicates that FA metabolism is critical in MS progression and should be explored as a potential target in MS therapy.

A common feature of these neurodegenerative diseases is the oxidative stress [148,149]. Cumulative oxidative stress induces cellular damage, impairment of the DNA repair system and mitochondrial dysfunction [150]. Mitochondrial FASN (mtFAS) synthesized lipoic acid, a potent antioxidant [151]. Lipoic acid downregulates proinflammatory redox-sensitive transduction processes such NF-κB translocation, thus diminishing the release cytotoxic cytokines and free radicals. Furthermore, lipoic acid reduces demyelization and axonal loss [152]. mtFAS deficiencies very specifically affect CNS function and lead to degeneration [23,153]. Thus, linoic acid metabolism is very promising as a therapeutic alternative in treating CNS disorders [151,154,155,156].

## 6. Conclusions

The findings summarized in this review highlight the role of FAs synthesized by glia cells in the regulation of the CNS. FAs can be both, beneficial and detrimental, depending on their degree of desaturation and/or elongation. Interventions targeting FA synthesis in glial cells could lead to potential therapeutic opportunity for several CNS diseases. Yet, further research must be carried out in order to unravel the contribution of glial FAs in disease pathology.

## Data Availability

No new data were created or analyzed in this study. Data sharing is not applicable to this article. Original figures created by A.V.G.C.
